# The effect of tibial component rotational alignment on clinical outcomes of mobile-bearing unicompartmental knee arthroplasty

**DOI:** 10.1186/s13018-023-03707-7

**Published:** 2023-03-20

**Authors:** Xiaoqiang Zhou, Chao Sun, Renjie Xu, Xiangxin Zhang, Xiao Yu

**Affiliations:** Department of Orthopaedics Surgery, The Affiliated Suzhou Hospital of Nanjing Medical University, Suzhou Municipal Hospital, Gusu School, Nanjing Medical University, Suzhou, 215000 China

**Keywords:** Unicompartmental knee arthroplasty, Mobile bearing, Tibial component rotational alignment, Knee Society Score, Forgotten Joint Score

## Abstract

**Background:**

The optimal tibial component rotational alignment in unicompartmental knee arthroplasty has not been defined. This study aimed to explore the effect of tibial component rotational alignment on the clinical outcomes of UKA.

**Methods:**

Clinical and follow-up data from 269 patients were retrospectively analysed. They were assigned into Groups A (− 5° to 0°), B (0°–3°), C (3°–6°) and D (> 6°) according to the external rotation of the tibial component to Akagi’s line. The Knee Society Score clinical (KSS-c), Knee Society Score function (KSS-f), Forgotten Joint Score (FJS) and postoperative complications at 2 years postsurgically were analysed.

**Results:**

The mean rotation of the tibial component relative to Akagi’s line in 269 patients was 4.56 ± 3.79°. There were 15, 84, 89 and 81 patients in Groups A, B, C and D, respectively. The postoperative KSS-c and KSS-f in Groups B and C were significantly higher than those in Group D. No significant differences in KSS-c and KSS-f were detected between Groups B and C. The postoperative FJS in Group B was significantly higher than that in Group C, which was significantly higher in Group C than in Group D. There were 5, 8 and 15 cases of postoperative knee pain in Groups B, C and D, respectively, and the difference was statistically significant.

**Conclusion:**

Tibial component rotational alignment is of significance to Oxford Phase III UKA in patients. External rotation of the tibial component by 0°–3° is optimal to achieve satisfactory clinical outcomes.

## Introduction

With the popularization of joint preservation treatment, the concept of a stepped care approach for knee osteoarthritis has been highlighted in clinical practice. Unicompartmental knee arthroplasty (UKA) is an effective surgical procedure for anteromedial osteoarthritis (AMOA). It preserves anatomic structures of the anterior cruciate ligament, lateral compartment and patellofemoral joint. Therefore, patients with anteromedial osteoarthritis of the knee have a wider range of motion (ROM), more natural characteristics of knee kinematics, a lower rate of complications and higher satisfaction after mobile-bearing or fixed-bearing UKA than after conventional total knee arthroplasty (TKA) [1–3].

Tibial component rotational alignment is of great significance in both TKA and UKA. Poor rotational alignment causes knee pain and stiffness, increases polyethylene wear, and impairs patellofemoral function, which significantly increases the risk of revision TKA [4]. Computed tomography (CT) scans have shown that the tibial component is mostly externally rotated compared with the posterior cortex of the proximal tibia [5, 6]. To date, tibial component rotational alignment assessed during UKA has not been fully analysed. Its potential influence on the clinical outcomes of mobile-bearing UKA is unclear. This study aimed to explore the optimal tibial component rotational alignment, thus achieving acceptable clinical outcomes of Oxford Phase III UKA in patients with anteromedial osteoarthritis of the knee.

## Methods

### Inclusion and exclusion criteria

Patients with anteromedial osteoarthritis of the knee who were surgically treated with primary Oxford Phase III, mobile-bearing, medial UKA in our centre were recruited.

Exclusion criteria: (1) surgical treatment with bilateral UKA; (2) extensive trauma or surgical treatment during the follow-up period; (3) severe chronic diseases; (4) loss of follow-up or incomplete clinical data.

### Data collection

A total of 361 patients with anteromedial osteoarthritis of the knee who were surgically treated with Oxford Phase III, mobile-bearing, medial UKA at the Affiliated Suzhou Hospital of Nanjing Medical University from January 2017 to December 2019 were retrospectively recruited. Ultimately, 269 eligible patients were allocated and classified into the internal rotation group (Group A) and external rotation groups (Groups B-D) based on tibial component rotational alignment. Patients in the external rotation groups were then arranged according to the angle in ascending order, and the case number was closed at cut-off values of 3° and 6°. We determined the internal rotation angle to be negative and the external rotation angle to be positive. Then, patients were assigned into Groups A (− 5° to 0°, *n* = 15), B (0°–3°, *n* = 84), C (3°–6°, *n* = 89) and D (> 6°, *n* = 81). The baseline characteristics, preoperative knee varus angle, and Knee Society Score (KSS) were recorded. This study was approved by the Ethics Committee of the Affiliated Suzhou Hospital of Nanjing Medical University.

### Perioperative procedures

Full-length standing anteroposterior radiographs were taken in every patient to assess the varus knee deformity. In patients with suspected ligamentous injury of the knee joint where the clinical assessment was unclear, MRI was performed to more accurately assess the extent of cartilage damage, and the anterior and posterior cruciate ligaments as well as the medial and lateral collateral ligaments were clearly identified before the surgery. Inflammatory arthropathy was excluded.

Doppler ultrasound examination of both legs was preoperatively performed to exclude cases of deep vein thrombosis (DVT). Moreover, colour Doppler echocardiography and chest CT were performed to exclude surgical contraindications. Intravenous administration of cefathiamidine 2 g and tranexamic acid 1.0 g was given 30 min before anaesthesia to prevent infection and operative haemorrhage, respectively.

**Fig. 1:**
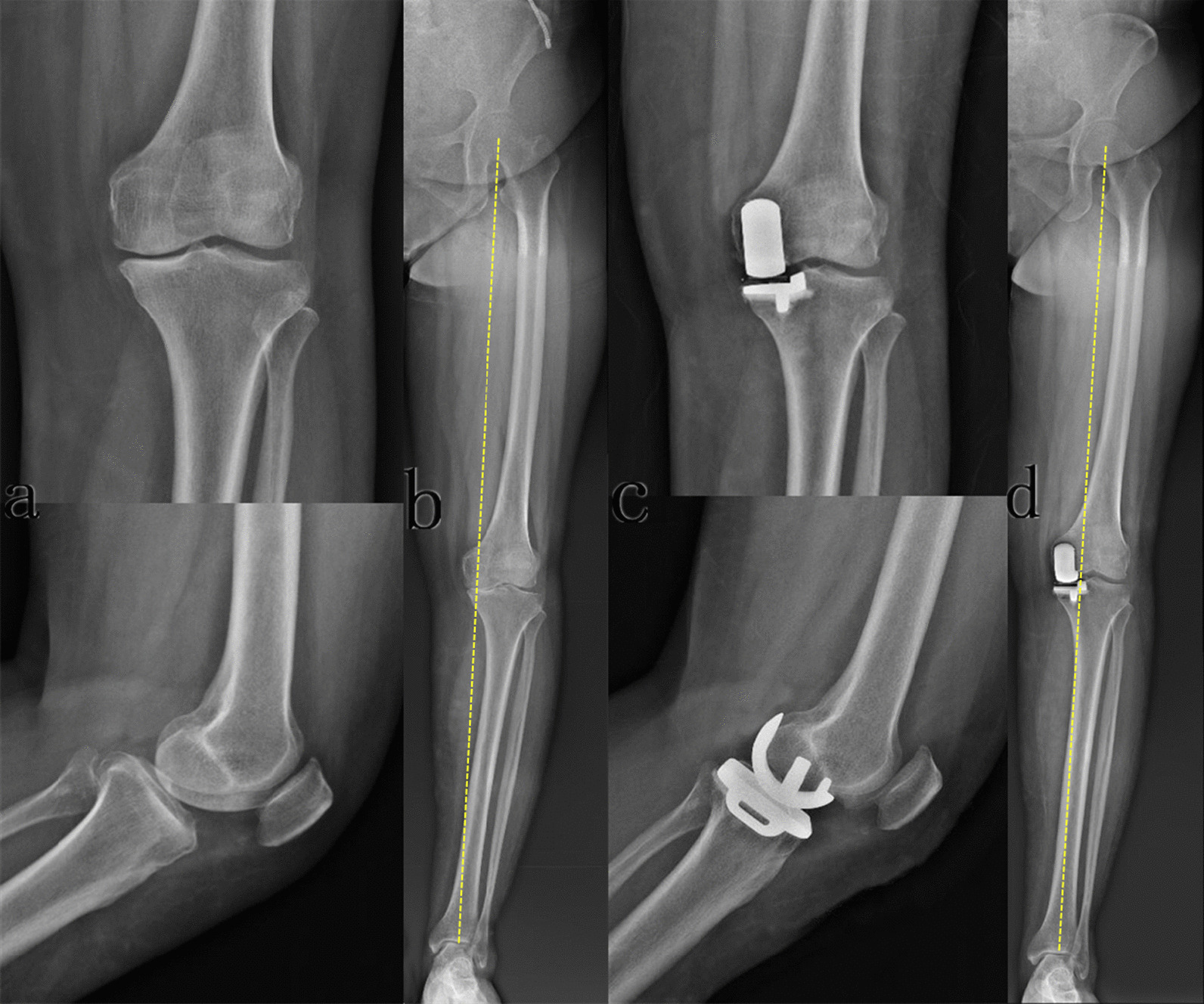
Representative images of mobile-bearing UKA. **a** Preoperative X-ray scans at the anteroposterior and lateral views suggested anteromedial osteoarthritis of the knee joint; **b** preoperative full-length standing anteroposterior radiographs suggested bone-to-bone contact in the medial compartment, with varus angles of 7.96°; **c** postoperative X-ray scans at the anteroposterior and lateral views suggested acceptable placement of the prosthesis; **d** postoperative full-length standing anteroposterior radiographs suggested acceptable placement of the prosthesis, with varus angles of 3.71°

UKA in all recruited patients was performed by the same team of surgeons using the mobile-bearing Oxford Phase III Partial Knee (Zimmer Biomet). After spinal anaesthesia, the affected lower limb was extended to the bedside using a standard bracket to maintain hip flexion at 45° and abduction at 30°. A pneumatic tourniquet was applied in UKA at a pressure of 40 kPa. Through the medial parapatellar approach, an incision was made from the medial margin of the patella to 3 cm at the distal end of the articular line. UKA procedures were performed based on the recommended manual of Oxford medial unicompartmental knee arthroplasty. Briefly, sagittal longitudinal osteotomy of the tibia was performed with the anterior superior iliac spine (ASIS) as the landmark. During the process of osteotomy, the patient's ankle was held by the left hand of the surgeon, slightly pulled down and rotated in a neutral position. The saw blade was placed close to the medial intercondylar ridge and the edge of the anterior cruciate ligament, which was pointed to the anterior superior iliac spine. The posterior edge of the tibial plateau was slightly extended, and potential damage to the posterior cortical bone was avoided. After prosthesis placement, the movement trajectory of the polyethylene component was checked to optimize tracking with the tibial component. The incision was sutured layer by layer. Representative images of mobile-bearing UKA are shown in Fig. 1.

Intravenous administration of cefathiamidine 2 g was given again at 8 h postoperatively, and tranexamic acid 2.0 g was given to reduce postoperative haemorrhage. Multimode analgesia involving ice packing and medications was given. Oral rivaroxaban 10 mg was given on the 1st day postoperatively to prevent DVT. Postoperative function rehabilitation was guided by the same team of experienced therapists, including active contraction of the quadriceps femoris and knee flexion and extension on the 1st day postoperatively and assisted walking 1–2 days later.

### Outcome measurement

**Fig. 2:**
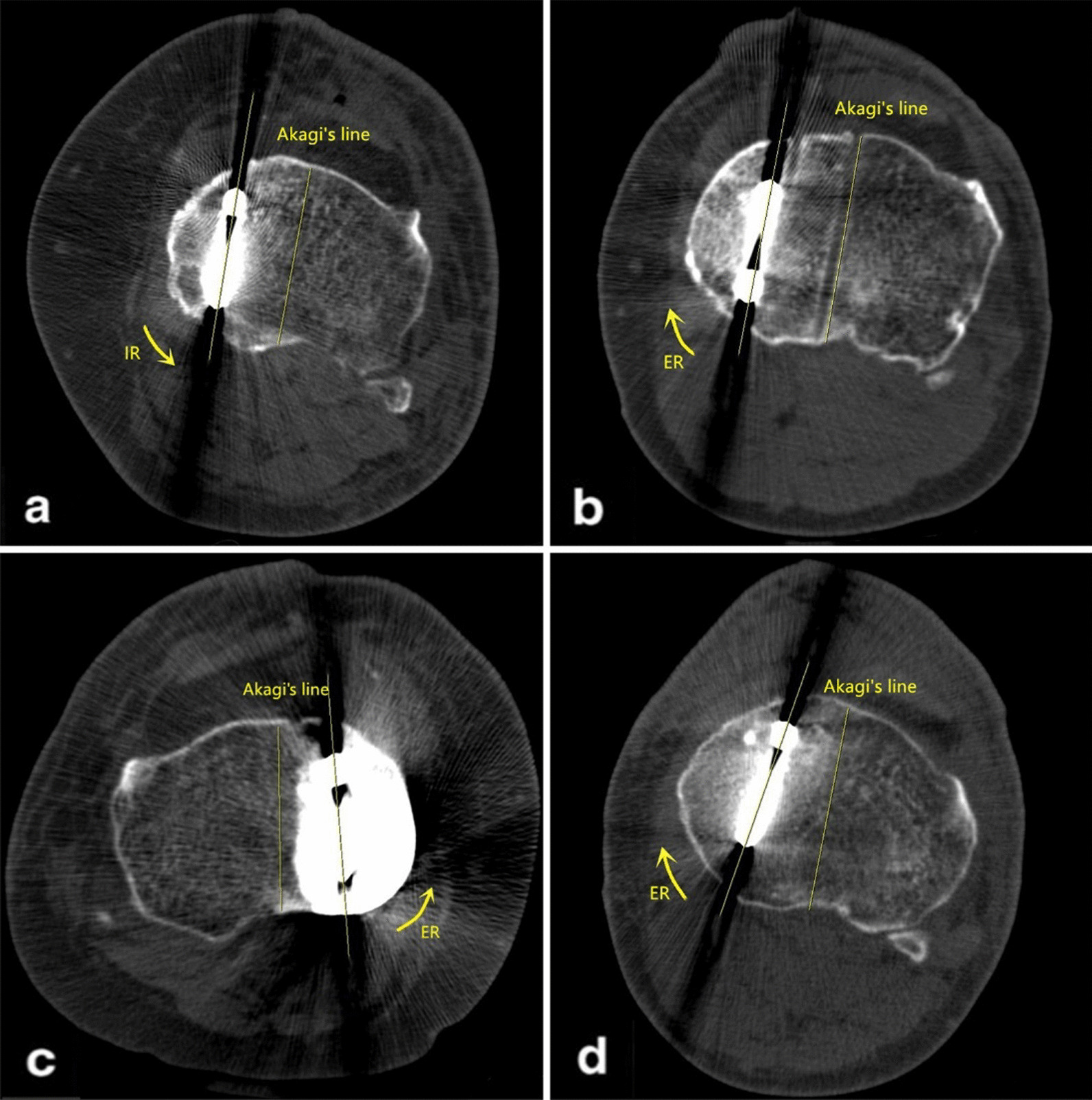
Tibial component rotational alignment relative to Akagi’s line. **a** Internal rotation (IR) at 0.89°; **b** external rotation (ER) at 1.02°; **c** ER at 4.12°; **d** ER at 8.76°

The postoperative knee varus angle was recorded by full-length standing anteroposterior radiographs. CT was performed to measure the tibial component rotational alignment. Briefly, the hip joint and knee joint were straightened to keep the patella positioned upwards, so the femur and shaft of the tibia were parallel to the horizontal plane, with the latter in the neutral position. Akagi’s line served as the rotational reference of the tibial component, which was defined as the line connecting the medial edge of the patellar tendon and the midpoint of the posterior cruciate ligament (PCL) [7]. All angles were measured by two experienced surgeons who were unaware of the content of this study, and the mean values were calculated. The external rotation of the tibial component relative to Akagi’s line was recorded as a positive value (Fig. 2).

All patients were followed up at the outpatient clinic at 1 month postoperatively, and they were examined by X-ray with anteroposterior and lateral views. Correct gait with walking aids and functional rehabilitation of the lower limbs were guided. The patients were later followed up at 3, 6 and 12 months and then once a year thereafter through telephone or at outpatient visits.

The KSS and Forgotten Joint Score (FJS) were recorded at 2 years postoperatively. The KSS consists of the Knee Society Score clinical (KSS-c) and Knee Society Score function (KSS-f), which was originally proposed in 1989 by the American Association of Hip and Knee Surgeons; the score simply assesses long-term postoperative recovery and overcomes the reduction of the Hospital for Special Surgery (HSS) scores due to age-related diseases. The FJS is a 0–100 scale that consists of 12 questions. It measures the ability of patients to forget about the joint prosthesis after treatment. The maximum score of 100 points indicates no pain and complete forgetting of the joint prosthesis.

Postoperative complications during the follow-up period were recorded, including knee pain, joint infection, prosthesis subsidence, progression of lateral compartment osteoarthritis and periprosthetic fractures. Knee pain with unknown causes was defined as the presence of knee pain from 3 months postoperatively, and the cause of trauma, joint infection, progression of lateral compartment osteoarthritis and prosthetic factors were excluded.

### Statistical analysis

Statistical analysis was performed using SPSS 25.0. Measurement data with a normal distribution, including age, body mass index (BMI), varus angle of the knee, KSS-c and KSS-f, were expressed as the mean ± standard deviation ($\overline{x} \pm s$), which were compared by one-way ANOVA with the least significant difference (LSD) test. Enumeration data, including sex and affected knees, were compared by the chi-square test. *P* < 0.05 was considered statistically significant.

## Results

### Baseline characteristics in patients with anteromedial osteoarthritis of the knee before Oxford Phase III UKA

**Fig. 3:**
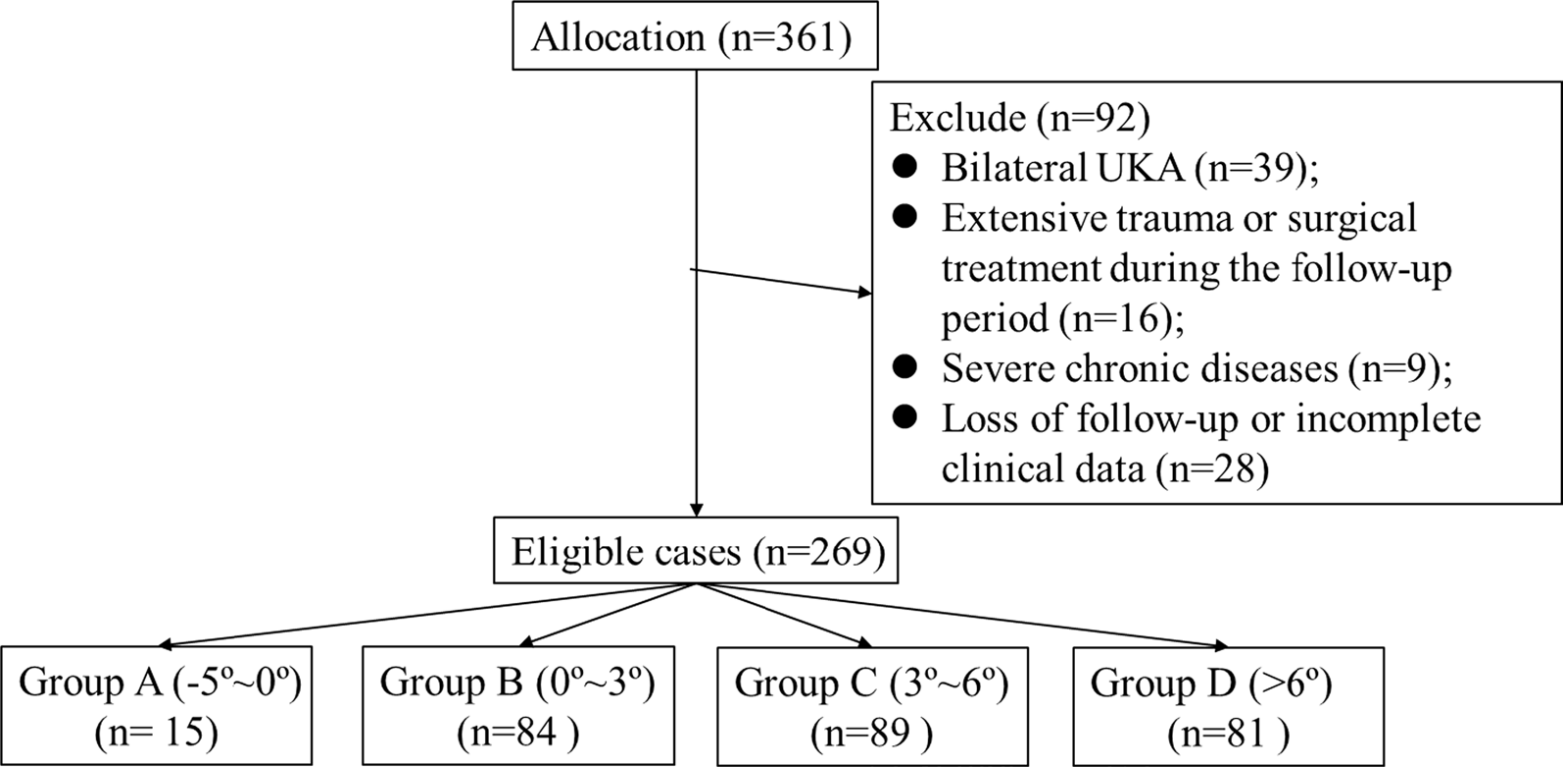
Flow chart of patient recruitment

A total of 361 patients with anteromedial osteoarthritis of the knee were initially recruited. After excluding those undergoing bilateral UKA (*n* = 39), suffering extensive trauma or being surgically treated during the follow-up period (*n* = 16), combined with patients with severe chronic diseases (*n* = 9) and loss of follow-up or lack of complete clinical data (*n* = 28), a total of 269 eligible patients were finally recruited for analysis (Fig. 3).

According to the rotation of the tibial component to Akagi’s line, only 15 patients were assigned to Group A. Data in Group A were not analysed and compared with those in the remaining groups due to the small sample size.

Baseline characteristics, including age, sex, BMI, affected knee, preoperative and postoperative knee varus angle, preoperative KSS-c and preoperative KSS-f, were comparable among Groups B, C and D (all* P* > 0.05, Table 1). The mean rotation of the tibial component to Akagi’s line was 4.56 ± 3.79° (− 4.87° to 13.90°), which was − (2.62 ± 1.18)°, 1.44 ± 0.88°, 4.66 ± 0.85° and 9.01 ± 2.48° in Groups A–D, respectively. All patients were followed up for an average of 45.25 (30–60) months.

**Table 1:** Baseline characteristics of patients with anteromedial osteoarthritis of the knee (*n* = 269)

	Group A^a^ (*n* = 15)	Group B (*n* = 84)	Group C (*n* = 89)	Group D (*n* = 81)	*F* value (*χ*^*2*^)	*P* value
Age (years)	67.60 ± 9.14	70.64 ± 9.23	69.24 ± 9.10	70.21 ± 8.79	0.551	0.577
Sex (male/female, *n*)	5/10	37/47	34/55	31/50	0.790	0.674
BMI (kg/m^2^)	26.92 ± 2.63	26.28 ± 2.61	26.16 ± 2.94	26.23 ± 2.59	0.044	0.957
Affected knee (left/right, n)	6/9	45/39	41/48	38/43	1.147	0.564
Preoperative varus angles (°)	9.69 ± 2.97	9.70 ± 3.50	9.38 ± 3.11	9.25 ± 2.94	0.446	0.641
Postoperative varus angles (°)	4.22 ± 1.06	3.94 ± 1.73	4.01 ± 1.67	3.79 ± 1.64	0.389	0.678
KSS-c (points)	55.13 ± 10.05	54.25 ± 7.89	53.44 ± 8.24	54.12 ± 8.85	0.239	0.788
KSS-f (points)	54.87 ± 9.68	52.79 ± 9.24	55.28 ± 9.75	53.80 ± 8.90	1.572	0.210

*BMI* body mass index, *KSS-c* Knee Society Score clinical, *KSS-f* Knee Society Score function

^a^Data in Group A were not included in the comparison due to the small sample size

### Clinical outcomes after Oxford Phase III UKA

The postoperative KSS-c in Groups B (85.73 ± 5.21 points,* P* < 0.001) and C (84.71 ± 6.55 points, *P* = 0.009) was significantly higher than that of Group D (82.21 ± 6.56 points), while no significant difference was detected between Groups B and C (*P* = 0.277). Consistently, the postoperative KSS-f in Groups B (87.04 ± 6.15 points, *P* = 0.034) and C (87.37 ± 6.12 points, *P* = 0.013) was significantly higher than that of Group D (85.04 ± 5.75 points), which was comparable between Groups B and C (*P* = 0.724). In addition, the postoperative FJS in Group B was significantly higher than that in Group C (76.75 ± 7.34 points vs. 74.02 ± 8.04 points, *P* = 0.020), which was significantly higher in Group C than in Group D (74.02 ± 8.04 points vs. 71.38 ± 7.60 points, *P* = 0.026) (Tables 2, 3).

**Table 2:** Clinical outcomes in patients with anteromedial osteoarthritis of the knee after Oxford Phase 3 UKA

	Group A^a^ (*n* = 15)	Group B (*n* = 84)	Group C (*n* = 89)	Group D (*n* = 81)	*F* value (*χ*^*2*^)	*P* value
KSS-c (points)	83.27 ± 6.69	85.73 ± 5.21	84.71 ± 6.55	82.21 ± 6.56	7.133	0.001
KSS-f (points)	86.20 ± 4.52	87.04 ± 6.15	87.37 ± 6.12	85.04 ± 5.75	3.633	0.028
FJS (points)	72.80 ± 6.79	76.75 ± 7.34	74.02 ± 8.04	71.38 ± 7.60	10.09	< 0.001

*UKA* unicompartmental knee arthroplasty, *KSS-c* Knee Society Score clinical, *KSS-f* Knee Society Score function, *FJS* Forgotten Joint Score

^a^Data in Group A were not included in the comparison due to the small sample size

**Table 3:** Pairwise comparison of clinical outcomes in patients with anteromedial osteoarthritis of the knee after Oxford Phase III UKA

	Group B versus Group C	Group B versus Group D	Group C versus Group D
KSS-c (*P* value)	0.277	< 0.001*	0.009*
KSS-f (*P* value)	0.724	0.034*	0.013*
FJS (*P* value)	0.020*	< 0.001*	0.026*

*UKA* unicompartmental knee arthroplasty, *KSS-c* Knee Society Score clinical, *KSS-f* Knee Society Score function, *FJS* Forgotten Joint Score

*P < 0.05

### Postoperative complications

During the follow-up period, there were 5, 8 and 15 cases of knee pain with unknown causes reported in Groups B, C and D, respectively. The incidence of postoperative knee pain was significantly different (*χ*^*2*^ = 7.217, *P* = 0.027) (Table 4). Patients with postoperative knee pain were managed by nonsteroidal anti-inflammatory drugs (NSAIDs), analgesia, symptomatic treatment, and functional exercise. Symptoms were relieved, and they were regularly followed up. A patient in Group D suffered knee pain and limited activity due to an accidental fall at 7 months postoperatively. Imaging evidence showed the dislocation of the polyethylene component. The patient was treated with revision UKA for replacing a thicker polyethylene component, presenting an acceptable postoperative recovery. Dislocation of the polyethylene component was not reported in the remaining groups. Although poor tibial component rotational alignment was achieved in some patients, they did not report uncomfortable symptoms and were continuously followed up. During the follow-up period, no cases of pulmonary embolism, symptomatic deep vein thrombosis, knee stiffness, joint infection, prosthesis subsidence, progression of lateral compartment osteoarthritis or periprosthetic fractures were reported.

**Table 4:** Postoperative complications after Oxford Phase III UKA (*n* = 269)

	Group A^a^ (*n* = 15)	Group B (*n* = 84)	Group C (*n* = 89)	Group D (*n* = 81)	*F* value (*χ*^*2*^)	*P* value
Knee pain (*n*)	0	5	8	15	7.217	0.027

*UKA* unicompartmental knee arthroplasty

^a^Data in Group A were not included in the comparison due to the small sample size

## Discussion

The present study found that the clinical outcomes of Oxford Phase III, mobile-bearing, medial UKA were linked with tibial component rotational alignment. Patients with anteromedial osteoarthritis of the knee suffered a poor prognosis after UKA at a minimum external rotation of 6°, while they achieved higher FJS and better subjective experiences at a maximum external rotation of 3°. We considered that the tibial prosthesis placed in a neutral position or mild external rotation at 0°–3° could yield satisfactory outcomes of UKA.

The femoral prosthesis of the mobile-bearing Oxford Partial Knee was designed with a spherical contact surface that could provide a relatively large deviation of internal or external rotation. Tibial osteotomy in the coronal plane is performed using surgical instruments, while in the sagittal plane, the surgeon removes a wedge-shaped portion of the tibia by hand. Therefore, the clinical outcomes of tibial osteotomy in the sagittal plane are significantly influenced by the surgeon's experience. At present, tibial component rotational alignment is difficult to control during surgery [8]. In our study, sagittal longitudinal osteotomy of the tibia was performed with the ASIS as the landmark. However, postoperative tibial component rotation varied greatly, with a mean external rotation of 4.56 ± 3.79° (− 4.87° to 13.90°), which was consistent with previous findings [5, 6]. Lee et al. [9] reported that the tibial component is mostly externally rotated compared with Akagi’s line (4.19 ± 3.72°) and the vertical line of the posterior cortex of the tibia (8.0 ± 6.1°) using the ASIS as the landmark during UKA. The cause of external rotation in UKA with ASIS as the landmark remains unclear, but, we believe, may be attributed to the following: First, the position of hip flexion and abduction in preparation for the surgery resulted in an elevated pelvis on the affected side, leading to internal displacement of the ASIS and external rotation of the tibial component. Second, it was difficult to determine a clear margin because the ASIS was beneath the sterile drape. Due to the small incision of UKA, the surgeon may intentionally externally rotate the tibial component to avoid damage to the PCL. Third, the knee joint was flexed during tibial osteotomy in the sagittal plane due to the pivot shift. As a result, the tibia was internally rotated compared with the femur, resulting in external rotation of the osteotomy line. In the present study, there were a small number of patients with postoperative internal rotation in Group A. They had acceptable outcomes, and complications were not reported during the follow-up period.

A slight mismatching of the femur and tibial component may lead to a high revision rate of UKA [10]. Improper tibial component rotational alignment eventually causes postoperative complications such as knee pain and stiffness, polyethylene wear and patellofemoral joint dysfunction, which significantly influence the clinical outcomes of UKA and quality of life [9]. Using Akagi’s line as the reference, the tibial prosthesis placed in mild external rotation at 0°–3° can yield satisfactory outcomes of UKA. Excessive external rotation of the tibial component may affect the trajectory and limit the motion of the polyethylene component, which further limits the flexion of the knee and postoperative ROM. Kamenaga et al. [11] consistently validated that the bearing may impinge on the lateral wall of the tibial component in the case of knee flexion above 60° if the tibial component is too much externally rotated. They also found that the tibial component rotation angle is negatively correlated with the Oxford Knee Score (OKS) at 2 years after UKA [12]. Iriberri et al. [5] retrospectively analysed the postoperative WOMAC pain score, KSS and visual analogue scale (VAS) scores in 88 patients receiving UKA, and they found that patients with better clinical outcomes have a smaller external rotation of the tibial component. More precisely, our findings highlighted a range of external rotation angles that yielded acceptable clinical outcomes of UKA.

It has been reported that great external rotation of the tibial component reduces bone coverage and causes overhang of the tibial component, which ultimately influences the clinical outcomes of UKA [5, 8, 12]. Chau et al. [13] demonstrated that an internal overhang of the tibial component at a minimum of 3 mm significantly reduces OKS. Therefore, excessive external rotation of the tibial component induces knee pain and reduces activities of daily living. In our study, we reported 28 cases of knee pain with unknown causes, which may be attributed to inflammatory responses in the medial collateral ligament (MCL) and surrounding soft tissues due to the overhang of the tibial component. Gudena et al. [14] consistently supported that the internal overhang of the tibial component by 2 mm increases the burden on the MCL, leading to postoperative knee pain.

To date, an optimal landmark for mobile-bearing tibial component rotational alignment is scant. The manual of the Oxford medial unicompartmental knee replacement recommended the alignment of ASIS during tibial osteotomy in the sagittal plane, which, however, lacks sufficient clinical evidence. It is inappropriate to determine the tibial component rotational alignment according to the distal anatomy. Lee et al. [9] found that the external rotation varies greatly during UKA with the ASIS as the landmark. Therefore, the use of ASIS is not recommended to guide tibial osteotomy during UKA. There are four types of landmarks guiding tibial rotation during UKA, including the femoral landmark, tibial plateau landmark, reference of the anatomical axis of tibia, and individualized osteotomy guide plate. The tibial component rotational alignment is determined by the angle of tibial osteotomy in the sagittal plane and the anatomic structure of the medial compartment, including the medial tibial spine, the edge of the anterior cruciate ligament and the medial femoral condyle. Kawahara et al. [15] analysed the efficacy of the medial wall of the intercondylar notch as the tibial rotational landmark, which was rotated ± 5° relative to Akagi’s line in 73.3% (33/45) of knee joints. However, their conclusion is stated regarding healthy knee joints, the anatomic structure of which differs from that of osteoarthritic knee joints. Tsukamoto et al. [16] suggested that the line connecting the medial border of the patellar tendon and the medial intercondylar tubercle is a more precise landmark for UKA than that of the medial intercondylar ridge in Japanese patients. Nevertheless, degenerative changes in the intercondylar wall and osteophyte hyperplasia may change the positional relationship of these landmarks and reduce the accuracy of tibial osteotomy. The optimal landmark for mobile-bearing tibial component rotational alignment still needs further analysis.

Several limitations in the present study should be noted. First, it was a retrospective study with a short follow-up period. The long-term clinical outcomes and survival of prostheses need to be further explored in large-scale, high-quality randomized clinical trials. Second, we focused on the effect of external rotation of the tibial component on the clinical outcomes of mobile-bearing UKA, and the conclusion should be validated in internal rotation and fixed-bearing UKA. Third, the correlation between tibial component rotational alignment and prosthesis coverage is unclear. Fourth, the large variation in tibial component rotational alignment with ASIS as the landmark has not been solved. We believe that individualized osteotomy guide plates, computer-aided localization or robotic operation are novel directions to enhance the accuracy of tibial component rotational alignment.

## Conclusion

Tibial component rotational alignment is of significance to Oxford Phase III UKA in patients with anteromedial osteoarthritis of the knee. External rotation of the tibial component by 0°–3° is optimal to achieve satisfactory clinical outcomes.
